# Experiences with regular testing of students for SARS-CoV-2 in primary and secondary schools: results from a cross-sectional study in two Norwegian counties, autumn 2021

**DOI:** 10.1186/s12889-023-16452-7

**Published:** 2023-08-15

**Authors:** Léa Franconeri, Sara Stebbings, Petter Heradstveit, Mia Johansen, Ragnhild Løken, Emily MacDonald, Liz Ødeskaug, Umaer Naseer

**Affiliations:** 1 Division for Infection Control, Norwegian Institute of Public Health, Oslo, Norway; 2 ECDC Fellowship Programme, Field Epidemiology path (EPIET), European Centre for Disease Prevention and Control (ECDC), Solna, Sweden

**Keywords:** SARS-Cov2, COVID-19, Educational settings, Students, Daily testing, Repetitive screening, Test-to-stay

## Abstract

**Background:**

To allow for normal school attendance during the COVID-19 pandemic, regular testing of students was introduced in the autumn 2021 in Norway to manage COVID-19 transmission. We mapped the experiences of five stakeholders (parents, students, school staff and administration, contact tracing teams) regarding the implementation of regular testing in primary and secondary schools in Oslo and Viken counties, to assess the acceptability through different indicators and improve future guidelines.

**Methods:**

A cross-sectional survey was conducted between October and November 2021 to explore experiences of implementation, compliance, satisfaction, difficulties, concerns, confidence in regular testing, quality of teaching and school attendance. Five stakeholder groups were invited to participate: contact tracing teams; school administrators and employees in primary, lower secondary, and upper-secondary school; students in upper-secondary school and parents of primary and lower secondary students. Bivariate analyses were performed for students, parents, and school employees groups. Descriptive analyses were done for contact tracing teams and school administrators.

**Results:**

Four thousand five hundred sixty-five participants were included in our study. School attendance increased for most of the students in primary and lower secondary schools in Oslo and Viken after the implementation of regular testing. Students across all school levels reported high testing compliance and satisfaction with the implementation. Compliance was significantly associated with an increasing number of weekly tests across all school levels up to two weekly tests. Contact tracing teams were less satisfied with the cooperation with the educational authorities compared to the school employees. Higher educational level of parents was significantly associated with decreased concern of their children getting infected at school after regular testing implementation. Concerned parents were more likely to keep children at home from school, to protect all household members from becoming infected. Lack of time and communication were reported as challenging factors to implementation.

**Conclusion:**

Compliance, satisfaction, and confidence in regular testing of COVID-19 were high among stakeholders. An acceptable testing regime for a future regular testing implementation would be a home-based, bi-weekly test. Increased awareness of the importance of school attendance, safety of regular testing along with good communication and role clarification should be prioritized for stakeholders involved in regular testing.

**Supplementary Information:**

The online version contains supplementary material available at 10.1186/s12889-023-16452-7.

## Background

Prolonged school closures during the COVID-19 pandemic have had a detrimental effect on students globally in terms of mental and physical health and the loss of critical resources for continuous learning [1, 2]. Throughout the course of the pandemic, schools around the world have sought to return to normal level of student attendance[3]. Evidence suggests that the use of proper mitigation strategies can reduce the risk for disease transmission between students and staff when reopening schools [1].

Screening programs are an example of a mitigation strategy that has been shown to reduce cases of COVID-19 in schools and thus reduce student-days lost to school closure or quarantine measures, as well as contributing to improved mental health and wellbeing [2, 4–6]. To allow for normal school attendance while simultaneously reducing COVID-19 transmission, Norway introduced regular testing (RT) of students in educational settings during the autumn of 2021. By screening children and adolescents in municipalities with high infection rates with rapid antigen tests, regardless of symptoms or exposure status, the strategy sought to reduce transmission rates across these age groups and reduce loss of in-person learning due to unnecessary quarantine.

The implementation of large-scale screening programs in schools like regular testing includes several stakeholders, ranging from users and facilitators to providers. In Norway, regular testing constituted a complex intervention where parents, students, teachers, school administrations and local health authorities played different part in the implementation process. In response to shifting epidemiological situations locally, Norwegian municipal health authorities could apply either: (1) *targeted temporary regular testing*, where testing was limited to one school or smaller units within schools (classes or age cohorts); or (2) *extended regular testing*, where whole age groups within municipalities were targeted for testing (e.g., all upper secondary schools). Depending on infection rates and test availability, it was recommended that participating students performed 1 – 2 tests per week. The national health authorities recommended testing to be performed at home before school in order reduce logistical burden, increase compliance, and maintain privacy. Municipal contact tracing teams provided schools with test kits and user instructions for further distribution to students or parents. Testing was voluntary, and there were no legal obligations to record the test activity and the test results. The test results did not appear in official statistics, except for positive tests that were confirmed with nucleic acid amplification test (NAT, e.g., Polymerase Chain Reaction) performed at municipal testing stations.

This study maps and analyses stakeholders´ experience with regular testing in primary, lower and upper-secondary schools, in Norway, during the COVID-19 pandemic. By providing policymakers and researchers with insights into how guidelines for regular testing strategies can be improved, our study seeks to support implementation of regular testing during future outbreaks or epidemics, especially within the context of infectious diseases transmitted through the respiratory tract. Some existing literature has already highlighted, focusing on user-perspective, that trust in health authorities, concerns regarding test safety and accuracy, potential consequences of positive test results and wider socioeconomic factors may influence on compliance with COVID-19 testing [7]. However, to our knowledge, few studies explore in detail how stakeholders’ experience regarding confidence, concerns, practical barriers, quality of teaching and school attendance interacts with regular testing practices and test adherence across stakeholders.

## Methods

### Study design

A cross-sectional study was conducted between week 42 (October 2021) to week 44 (November 2021) in Oslo and selected municipalities in Viken county (Lørenskog, Ullensaker, Fredrikstad, Lillestrøm, Drammen, Bærum and Asker) known to have implemented regular testing. We distributed five online surveys to match each subset of the target population.

### Participants

The study sample was composed of five subsets: contact tracing teams (regular testing providers), school administrators and staff (regular testing facilitators in primary, lower secondary, and upper-secondary schools), students (regular testing users in upper-secondary > 15 years of age), and parents (proxy regular testing users for students in primary and lower secondary > 5 and < 15 years of age). Participants met the inclusion criteria if they were working in a municipality or school where regular testing was implemented, were parents who had children who participated in regular testing or were students who participated in regular testing at their school.

### Research procedure

We used *Nettskjema*, a secure electronic survey platform to create and distribute the surveys via email. The survey link was sent to municipal chief physicians, primary and lower-secondary school authorities at the municipal level, and upper-secondary authorities at the regional level, who then forwarded the survey to relevant management teams of local contact tracing teams and schools. The school management would then forward the survey invitation to the remaining sample units (students, parents, and school staff). All questionnaires were developed in Norwegian while the survey for parents were translated into Arabic, Polish, Somali, and English to facilitate for increased participation and representation across different language groups.

By using this distribution pattern, we did not know the number of participants at endpoint who received the survey invitations and are therefore unable to calculate sample size and response rate. Electronic informed consent was obtained from all participants answering the survey. The study was exempted from formal ethical approval by the Regional Ethics Committee of South-East Norway 08.10.2021 (reference number: 355036) due to being a non-sensitive and anonymous survey.

### Outcome measures

Seven key outcomes for the survey were collected: compliance (students and parents responding on behalf of their children only), satisfaction, confidence, difficulties, concerns related to the implementation of regular testing, quality of teaching (school staff only) and increase in school attendance (students and parents responding on behalf of their children only) (Table 1).

**Table 1:** Description of outcome measures

• Test compliance was measured by (” How many of the tests did you usually complete”, where responses ranged from 1-None of the tests to 5-All the tests). Compliance was defined as performing most of the tests or all the tests
• Satisfaction was measured in relation to organization, communication/information, and level of cooperation during the implementation of regular testing, where responses ranged from 1-Dissatisfied d to 5-Very satisfied. Satisfaction was defined as satisfied or very satisfied
• Confidence was measured as (“How much confidence do you have in the health authority’s decision to implement regular testing instead of quarantine for children”, where responses ranged from 1-Very little to 4-A lot). Confidence was defined as some or a lot
• Difficulties was measured as (“Have one or more of the following factors made it difficult for you/your child to participate in regular testing”, where participants could select all that applied from No difficulties, Access to testing equipment, Communication/information, Training, Time usage, Personnel, and Motivation. Subjects including answers with No difficulties were put in the category of “no difficulties reported” regardless of selecting additional factors
• Concerns were measured in relation to the self-experienced risk of acquiring infection at school by one domain (“Since the introduction of regular testing, I have been concerned that I/my child will become infected at school”, with responses ranging from 1-Completely disagree to 5-Completely agree). Concern was defined as agree to some extent or completely agree
• Quality of teaching was measured as (“How has regular testing impacted the quality of teaching in the classes you have taught?”, where responses ranged from 1-Poorer quality to 5-Improved quality. Improved quality was defined as some improved quality to improved quality and vice versa for poorer quality
• Attendance was measured as (“After the implementation of regular testing, I/my child has had better attendance at school”, where responses ranged from 1-Completely disagree to 5-Completely agree). Attendance

At the end of each survey an optional free text question was added for respondents to elaborate on experiences linked to regular testing.

### Statistical analysis

Data obtained from the surveys was subject to analysis using R (version 4.1.1) and Stata (version 16.0). The statistical significance was considered at *p* < 0.001. Bivariate analysis using Chi-squared tests of independence or Fisher exact tests were used to assess non-random differences between compliance (students and parents), satisfaction (students, parents, and school staff), confidence (parents and school staff), concerns (students, parents, and school staff) and school attendance (students and parents) for the variables of interest. No statistical analysis was performed for contact tracing teams and school administrations due to small sample sizes and only descriptive data is presented. Municipalities were grouped together, analyzed, and presented throughout the study at county-level (Oslo and Viken).

## Results

### Study population

**Fig. 1:**
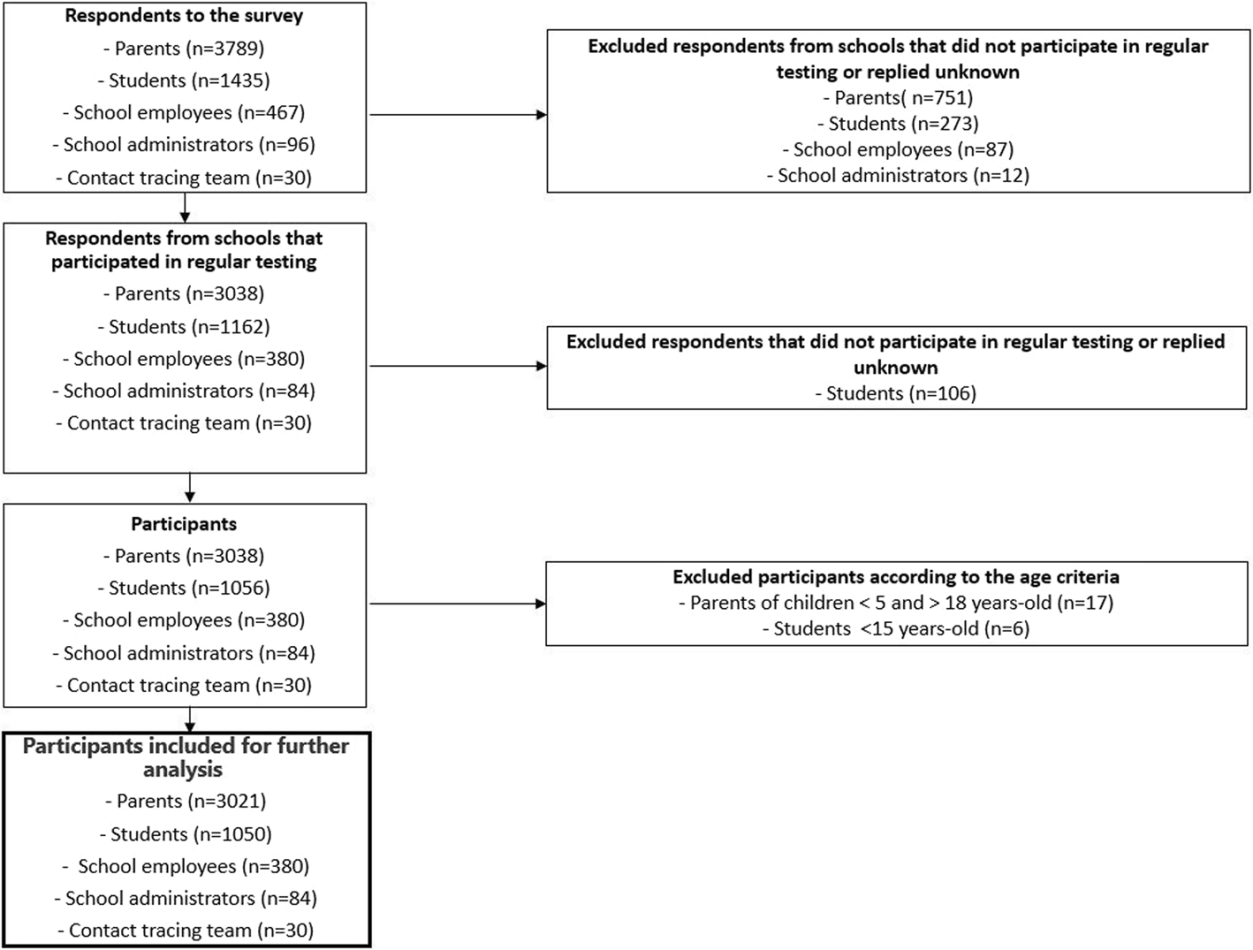
Flowchart of the study population selection process

A total of 5 817 participants completed the questionnaires from the two counties Viken (municipalities: Asker, Bærum, Lillestrøm, Drammen, Lørenskog and Fredrikstad) and Oslo, across the different subsets; respondents were excluded based on our inclusion criteria and the remaining 4 565 were included for further analysis (Fig. 1, Additional file 1).

Characteristics of school employees, students, and parents are shown in Additional file 2. Most of the respondents were female, including school employees (70%), students (62%), and parents (74%), with a median age of 46, 17, and 46 years-old respectively. The school employees were categorised as teachers (*n* = 309, 81%), other personnel in student-oriented work (child welfare workers, physical therapist, social workers) (*n* = 46, 12%), and other (librarians, janitors, and such) (*n* = 25, 7%), working at upper secondary schools (*n* = 186), lower-secondary schools (*n* = 129) and primary schools (*n* = 82) (percentage not calculated as multiple answers were possible). Students were predominantly in their first or second year of upper-secondary school education (respectively, *n* = 507, 48% and *n* = 426, 40%), while most of the children with parents responding on their behalf were in primary (*n* = 1654, 55%), lower-secondary school (*n* = 1358, 45%) or in other unknown school grade, which was not specified but within public institutions (*n* = 9, < 1%).

### Difficulties in implementation

Communication and information flow were the most reported difficulties related to regular testing among contact tracing teams (27%), while time usage was the main difficulty reported by school administrators (28%), school employees (25%), students (44%) and parents (23%) (Table 2). Re-packaging and distributing the test kits was particularly pointed out as time-consuming by the school administrators and employees. The second most challenging factor reported by the school administrators and school employees, respectively, were lack of personnel (17%) and issues related to communication and information flow, regardless of whether it came from public health authorities or municipality (19%). Students reported motivation (32%) as a challenging factor while access to testing equipment (18%) was reported by parents.

**Table 2:** Common difficulties in the implementation of regular testing in the autumn of 2021 by contact tracing teams, school administrators, school employees, students, and parents in Oslo and Viken

Sample group					
	**Contact tracing teams, *N* = 30**	**School administrators, *N* = 84**	**School employees, *N* = 380**	**Students, *N* = 1050**	**Parents, *N* = 3021**
**No difficulties reported**	7 (23%)	34 (35%)	169 (45%)	637 (61%)	2339 (77%)
**Challenging factors**^**a**^					
Access to testing equipment	5 (10%)	13 (10%)	45 (10%)	95 (25%)	148(18%)
Communication/Information	13 (27%)	19 (15%)	88 (19%)	66 (18%)	71 (8.4%)
Training	5 (10%)	7 (5%)	68 (14%)	44 (12%)	42 (5%)
Time usage	5 (10%)	37 (28%)	118 (25%)	164 (44%)	197 (23%)
Personnel	2 (4%)	22 (17%)	44 (9%)	-	-
Motivation	-	-	-	119 (32%)	-
Other factors^b^	19 (39%)	10 (8%)	28 (6%)	49(13%)	633 (75%)

^a^The respondents could select more than one response to this question

^b^Among individuals who reported a difficulty, other factor included free-text option, negative reactions from family/friends/employees/ students undergoing testing, physical discomfort, and mental discomfort. These variables were not displayed separately due to small numbers

### Compliance

An overall high degree of compliance to regular testing was reported by parents on behalf of their children in primary and lower-secondary schools (*n* = 2801, 93%) and by students in upper-secondary schools (*n* = 970, 92%) (Additional file 3). Compliance to test appeared to increase with the age of the parents and the grade level of the children in school (*p* < 0.001).

A better compliance was highlighted for a frequency of two tests per week compared to one or three weekly tests (*p* < 0.001). Furthermore, the administration of tests within the school setting seemed to be linked to increased compliance among primary and lower secondary school children, despite the majority of parents conducting tests at home. The parents who were confident in the health authorities were more likely to test (*p* < 0.001) as well as the upper secondary school student who adhered to general Infection and Prevention Control (IPC) measures (*p* < 0.001). Conversely, a lack of training and time among parents, as well as a lack of motivation and time among upper secondary school students, were associated with reduced compliance in conducting tests (*p* < 0.001).

### Satisfaction

Contact tracing teams were moderately satisfied (≥ 54%) regarding their cooperation with the school administration and educational authorities. However, in Oslo, contact tracing teams reported dissatisfaction with the information received, the availability of testing equipment and the clarity about role definitions, during the initial implementation phase of regular testing (Additional file 4).

School administrators reported a high degree of satisfaction (> 80%) in their cooperation with the political authority of their municipality, regarding the information flow, availability of testing equipment and guidance on how to perform the tests.

School employees reported a notable level of satisfaction in their cooperation with the school administration (> 70%) particularly in terms of access to updated information and to testing equipment. Among personnel in student-oriented work (child welfare workers, physical therapists, social workers), satisfaction with regular testing reached 74%, compared to 68% among other personnel (librarians and janitors) and 67% among teachers. In both counties, the relationship between the school employees and the parents or the students was unaffected by implementation of regular testing (> 75% in both groups).

However, despite the level of satisfaction, most of the contact tracing teams, school administrators and school employees reported an increase in workload following the implementation of regular testing (Table 3). Responses indicated that the workload surge was particularly pronounced during the initial phase of implementing regular testing. School administrators and employees identified the re-packaging and the distribution of test kits, along with limited personnel, as the primary factor contributing to this increased workload. Lastly, most of the parents of children in primary and lower secondary schools, as well as students in upper secondary schools expressed their satisfaction with the implementation of regular testing in both counties (Table 4).

**Table 3:** Regular testing and the impact on workload by contact tracing teams, school administrators and school employees in Oslo and Viken

	**Contact tracing teams**		**School administrators**		**School employees**	
	Oslo, *N* = 24^a^	Viken, *N* = 6^a^	Oslo, *N* = 33^a^	Viken, *N* = 51^a^	Oslo, *N* = 133^a^	Viken, *N* = 247^a^
Increased	8 (33%)	3 (50%)	18 (55%)	33 (65%)	69 (52%)	134 (54%)
Reduced	15 (63%)	2 (33%)	0	4 (8%)	2 (1%)	4 (2%)
Unchanged	1 (4%)	1 (17%)	15 (45%)	14 (27%)	62 (47%)	109 (44%)

^a^n (%)

**Table 4:** Satisfaction levels among parents of primary and lower secondary school students and upper secondary students in Oslo and Viken

	**Parents (primary and lower-secondary), *N* = 3021**			**Students (upper-secondary), *N* = 1050**		
**Satisfaction in relation to:**	Oslo, *N* = 1782^a^	Viken, *N* = 1239^a^	*p*-value^*^	Oslo, *N* = 59^a^	Viken, *N* = 991^a^	*p*-value^*^
**Information provided by schools**			**0.8**			**0.5**
Satisfied	1502 (84%)	1025 (83%)		44 (75%)	638 (64%)	
Not satisfied	79 (4%)	52 (4%)		3 (5%)	79 (8%)	
Unknown	201 (11%)	162 (13%)		12 (20%)	274 (28%)	
**Information provided by municipalities**			**< 0.001**			**-**
Satisfied	1000 (56%)	864 (70%)		-	-	
Not satisfied	176 (10%)	86 (7%)		-	-	
Unknown	606 (34%)	289 (23%)		-	-	
**Implementation of regular testing**			**0.2**			**0.6**
Satisfied	1443 (81%)	1031 (83%)		43 (73%)	633 (64%)	
Not satisfied	109 (6%)	64 (5%)		2 (3%)	59 (6%)	
Unknown	230 (13%)	144 (12%)		14 (24%)	299 (30%)	

^*^Pearson's Chi-squared test, Fisher's exact test

^a^n (%)

### Confidence

Contact tracing teams expressed a high level of confidence in regular testing, as did the school administrators (Additional file 5). Parental confidence was reported at approximately 70%, with parents having higher educational levels displaying greater confidence compared to those with lower educational backgrounds (48% among individuals with ≥ 4 years at university level). Additionally, among school employees, we noticed a slight increase in confidence levels with age. To conclude, employees working in upper secondary schools showed higher confidence levels (83%, *n* = 155) compared to those working in primary and lower secondary schools (76%, *n* = 62 and *n* = 98).

### Concerns

Following the implementation of regular testing, there was no significant increase in overall concerns among school administrations, school employees, and parents regarding their own or their children's potential infection with SARS-Cov2 (Additional files 6, 7 and 8). Also, most of the students in upper secondary school reported reduced worries of becoming infected at school after regular testing was introduced. No association were found between socio-demographic characteristics such as parental and student age, as well as the students' school grade, and the level of concern. However, our results showed an association between educational level and concern among parents (*p* < 0.001), where parents having a university background were less concerned about their child's risk of infection at school (Additional file 9). Conversely, parents who reported being concerned were more likely to keep their children at home, away from school (*p* < 0.001) and indicated a higher degree of absence in extracurricular activities of their children (*p* < 0.001) (Additional file 10). Moreover, our results indicated that students who reported a lack of concern about contracting the virus at school were more likely to participate in testing.

### Quality of teaching and attendance

Overall, school employees reported that the introduction of regular testing did not impact the quality of teaching. Moreover, digital lessons were not usually introduced, even though, in both counties, less than 20% of the employees reported digital home-schooling for approximately six days or more in total during the autumn of 2021. Student self-reported attendance increased overall for both primary and lower secondary schools, as well as for upper secondary schools, following the introduction of regular testing, even if around 30% of the respondents remained neutral (Table 5). Also, when comparing the groups by school attendance (Additional file 11), an increase in attendance was reported by the parents being compliant to testing, confident in public health authorities and satisfied with information provided by school, municipalities or about the organisation of regular testing (*p* < 0.001). Conversely, the parents who reported a lack of time mostly reported no change in school attendance. Students, on the other hand, reported increased school attendance mainly when they were satisfied with the organisation of regular testing, or the information provided by their schools (*p* < 0.001).

**Table 5:** Self-reported attendance among students in primary, lower secondary and upper secondary school students in Oslo and Viken after the implementation of regular testing

	**Parents (primary and lower-secondary), *N* = 3 021**			**Students (upper-secondary), *N* = 1050**	
**My child’s attendance at school has increased**	Oslo *N* = 1782^a^	Viken *N* = 1239^a^	**My attendance at school has increased**	Oslo *N* = 59^a^	Viken *N* = 991^a^
Agree	853 (48%)	738 (59%)	Agree	28 (47%)	422 (42%)
Disagree	297 (17%)	154 (12%)	Disagree	9 (15%)	158 (16%)
Neutral	632 (35%)	347 (28%)	Neutral	22 (37%)	411 (41%)

^a^n (%)

## Discussion

This study investigated experiences with the implementation of regular testing in primary, lower and upper-secondary schools among involved stakeholders in Oslo and Viken. Our results showed a high level of test compliance across all school levels, as well as an overall high level of satisfaction and confidence across the groups surveyed. However, certain challenges were highlighted, such as lack of communication and information, as well as role clarification among stakeholders, and factors that can potentially influence compliance.

### Quality of teaching and attendance

Regular testing was implemented with the goal of maintaining normal school attendance and reducing student days lost to school closure or quarantine. Our study supports that this was achieved as the results indicate that regular testing did not affect the quality of teaching, and digital lessons were usually not implemented during the study period. Self-reported attendance overall was high among students, although with some differences between counties. Regular testing may have prevented unnecessary absences and maintained important levels of in-person teaching, which is in line with a recent study from France showing that regular testing could reduce lost school days by up to 80% compared with reactive class closures [8].

### Compliance

Test compliance was above 90% across all school levels and results suggested that 2 tests per week led to a better compliance than one test per week. Modelling studies have found that testing twice a week can reduce the attack rate and transmission of COVID-19 [4, 9]. In another study modelling the impact of different testing regimens in the university setting, the authors suggested that higher frequency of testing could further reduce the size of the epidemic [5]. Results from primary and secondary schools in our study suggest that increasing testing frequency to three tests per week may negatively impact compliance.

Our results indicate that test compliance was lower for parents with children in primary schools compared to students in upper secondary schools, and higher when the tests were performed at schools. This is likely associated with the challenges linked to testing younger children, who require more assistance, making school testing a more convenient alternative. Additionally, both lack of time and training were reported as significant barriers for testing among parents. A review on behaviour related to COVID-19 testing showed that the age and the dependency of the child could impact test adherence or the willingness to test [10]. This could be related to fear of the consequences of a positive test, such as loss of income if the parents had to stay at home with their sick child. Our results also support the previous findings from a German study, where parents of primary school children appreciated home testing, but still preferred their children to be tested at school [6]. However, it should be noted that a positive test may be associated with social stigma, which in turn could lead to unwillingness to participate in regular testing and result in a detrimental effect on the mental health of children. Although school testing can positively impact on testing compliance, we still consider the levels of compliance related to home testing to be sufficient, as well as being a better option to avoid stigmatisation of children and increased burden on school staff. Our results did not show any significant association between testing compliance and the school grade among students in upper secondary school, although a previous study has suggested that compliance can be lower among students in higher school grades [11]. Additionally, no significant difference in compliance was observed for upper secondary school students performing the test at home or at school, possibly indicating that older children are more able to administer the tests on themselves. However, we found that students in upper secondary school who were compliant to testing had better adherence to general IPC measures, suggesting that better understanding of the importance of the recommended preventative measures leads to better compliance rates. Significant barriers associated with compliance were reported to be lack of motivation and lack of time. This highlights overall the importance of communication adapted to different social and age groups in the setting of infection control. A Swiss prospective study among 22-year-old students showed that non-compliance, especially in relation to hygiene-related measures, was more prevalent in males, and in individuals with higher education, higher socio-economic background, and without foreign origin [12]. We did not find any difference regarding students’ compliance as a function of gender or with at least one parent of foreign origin in our study (Additional file 12). We found that compliance and trust were positively associated, and parents who had confidence in regular testing as a safe alternative to quarantine reported higher compliance. Personal experience with regular testing also impacted compliance, and children who previously had a negative experience with regular testing appeared to be less compliant.

### Satisfaction

Although overall satisfaction levels were high across the surveyed groups, information and role clarification was less clear among contact tracing teams and school administrations. Furthermore, more than 50% of the contact tracing teams, school administrations and school employees reported increased workload after the implementation of regular testing. However, contact tracing teams in Oslo reported a reduced workload, highlighting differences in the local administration of regular testing in Oslo compared to Viken county. Lower satisfaction levels were linked to poor communication and lack of role clarification, particularly during the implementation phase of regular testing, and in relation to re-packaging of test-kits that was required from the school administrations. This highlights the need for better communication between facilitating stakeholders and health authorities, and early clarification of roles before implementation of IPC measures.

### Confidence

Trust in public health authorities has a significant impact on public attitudes and behaviours, which in turn affects how people adhere to health recommendations [7]. Our results suggests that contact tracing teams, school employees and parents had confidence in the health authority’s decision to implement regular testing in schools as an alternative to quarantine. Employees working in upper secondary schools were inclined to higher confidence levels compared to employees working at primary and lower secondary schools. This could be due to the closer interaction between staff and students at lower levels of schools, and thus impacting on their perceived risk of infection while at work. Also, age and educational level were seen as a contributing factor for confidence, as it increased with the educational level of the parents and with the age of the school employees. This reaffirms the findings of a previous study regarding trust in Public Health Authorities in the United States, where highly confident individuals were likely to be older, have higher educational level and greater income. Confident parents were more likely to have children in higher school grades, a pattern seen as well across the school employees working in higher school grades, showing a higher confidence when working with slightly older children [7]. A Norwegian study showed that trust in government was higher among women, individuals with higher education, older age and individuals working in the public sector, which also corresponds with our findings [13].

### Difficulties in implementation and concerns

Practical obstacles as well as attitudes and behaviour can influence implementation and adherence to IPC measures. School administrators, school staff, students, and parents alike pointed out lack of time as the main challenge for implementation and adherence to regular testing. School administrations and staff highlighted re-packaging and distribution of test-kits as particularly time consuming. This obstacle should be possible to overcome through more efficient systems of re-packaging and distribution. The lack of time reported by parents and students in relation to performing the tests at home could be addressed by testing at schools. However, the logistical burden on schools and privacy concerns for children cannot be overlooked. Among upper secondary school students, test compliance was associated with concerns for acquiring COVID-19, as students who were less worried of acquiring infections reported slightly higher levels of compliance. It is unknown whether the concern for acquiring infection is related to health, stigma, or other applicable IPC measures due to a positive test. Among parents, our findings indicate that concerns for acquiring infection during regular testing are associated with lower levels of educational background. Although regular testing aims to increase school attendance, some parents report to have kept their children home from school due concerns about exposing the children, themselves, or the household to COVID-19. For contact tracing teams, the main challenge in implementation was related to communication and flow of information, especially with the health authorities. Overall, these findings indicate the need for regular testing to integrate tailored communication strategies. Firstly, accompanying regular testing with efforts that address students’ concerns regarding the consequences of positive test might reduce stigma and fear and, in turn, help increase compliance. Secondly, addressing parents’ concerns regarding the effectiveness of regular testing and strengthen awareness of in-person learning might help to reduce student-days lost to concern among parents and avoid enhancing socio-economics differences in school attendance. Finally, clarifying communication lines and areas of responsibility in relation to stakeholders and health authorities might ease implementation on the part of contact tracing teams.

### Strengths and limitations

**Fig. 2:**
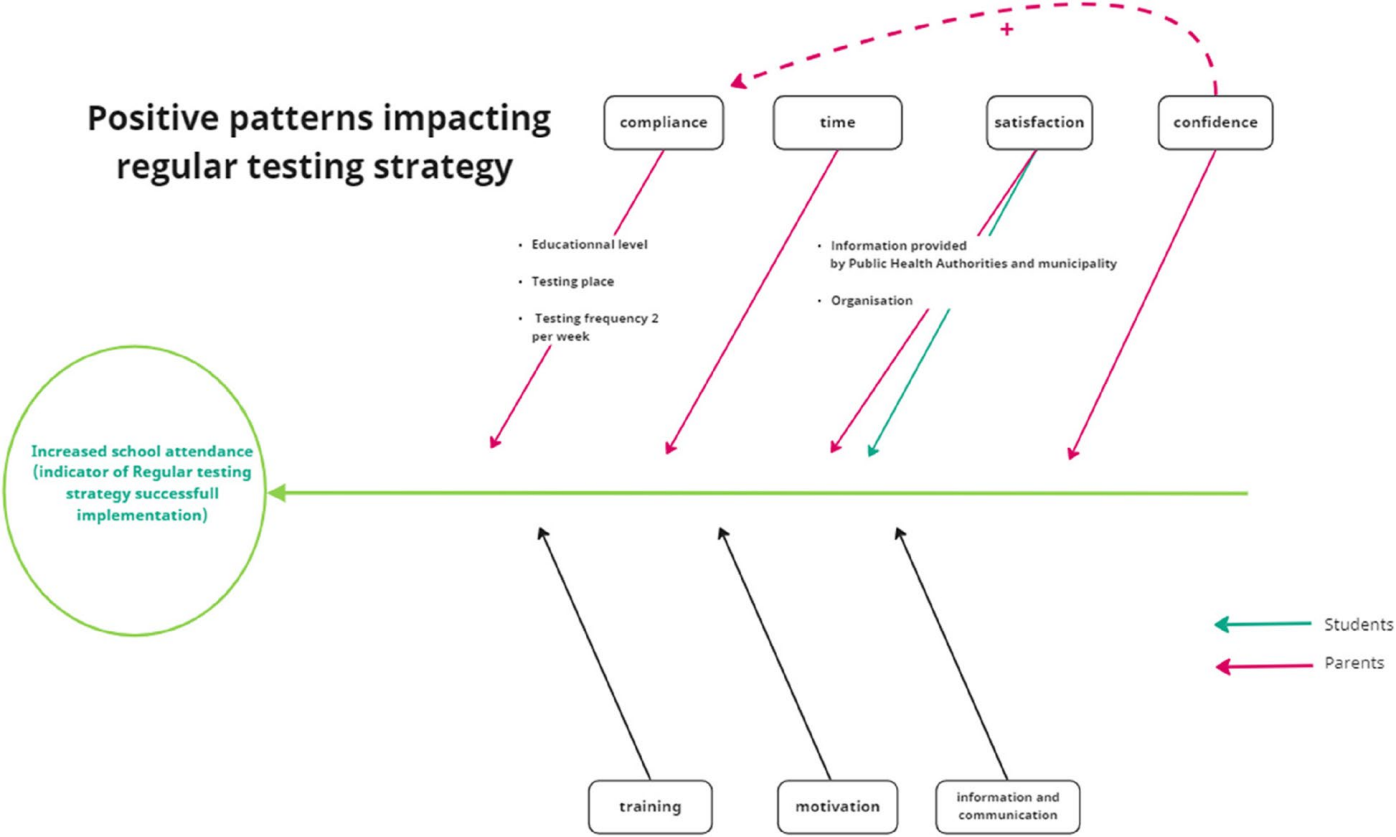
Modified fishbone diagram on the patterns impacting the increase in school attendance, chosen as a main indicator for a successful implementation of regular testing. Colored arrows represent a significant relationship between a pattern and increase in school attendance for users' specific groups (Additional file 11)

This study is one of few regional studies conducted to investigate experiences and compliance to regular testing of COVID-19 in educational institutions from different stakeholders. This study did not aim to compare the impact of regular testing versus quarantine on infection rates in school settings. By including the perspective of providers, facilitators, and users, we have been able to both extract the experience of each group and examine how these experiences interact with one another. Using an indicator to measure if the regular testing strategy was successful (increase of school attendance), we were able to identify the patterns which could lead to this success (Fig. 2, Additional file 11). In addition, we facilitated for increased participation among the largest foreign language groups in Norway by translating the survey for parents into Arabic, English, Polish, and Somali.

However, there are some important limitations in our study to be addressed. Firstly, parents were used as proxy for their children in primary and lower secondary school. Secondly, regular testing was implemented over a longer period throughout the autumn of 2021 before being gradually phased out. Although some free text answers indicated that the implementation improved over time, it was not possible for us to examine how experiences developed over time due to the cross-sectional design of the study. Nonetheless, we carried out the study shortly after regular testing was phased out in the schools so that participants had more accurate recollection. Thirdly, by using an electronic survey where the included municipalities were used as an intermediate for further distribution, our responses could have been prone to selection bias induced by a voluntary selection design. Individuals who volunteer to participate in studies may not be representative of the general population. As the responses are based on self-reporting, it is also possible that some of answers are subjected to reporting bias. Furthermore, users with low digital skills could have found it difficult to participate [14] and some individuals may have been discouraged from participating due to the length or the wording of the survey questions. Finally, as the sample size was not calculated before the survey was launched, we were not able to compute a response rate and size our sample to be representative before launching the survey. However, assuming a target population of around 185 441 [15, 16], with a sample proportion of 50% and a margin of error of 5%, the minimum necessary sample size would be 385 individuals. With around 4000 responses included in the analysis, it appears that the actual number of participants in the study was greater than the estimated required sample size.

## Conclusion

This study found that the levels of compliance, satisfaction, and confidence regarding regular testing of COVID-19 were high among stakeholders involved with regular testing in Norwegian schools. The findings suggested that a bi-weekly testing regime carried out at home could maintain sufficient test compliance while reducing burden of implementation. However, to increase compliance with regular testing and further reduce student-days lost to COVID-19, regular testing strategies should seek to strengthen stakeholders’ awareness of in-person learning and increase confidence in the safety of regular testing as a sound alternative to quarantine or closure. Moreover, to strengthen implementation, regular testing strategy leaders should seek to clarify operational roles and facilitate for good communication flow among stakeholders such as health authorities, contact tracing teams and school administration. Policymakers should also ensure that information and training material are available among users, to reduce concerns, increase confidence, increase awareness of the benefits of regular testing and school attendance. These findings should help to achieve the objectives of regular testing, in high risks settings, for example in future epidemics, and more specifically due to respiratory pathogens.

### Supplementary Information

**Additional file 1. **Number of participants included from the different groups by county of residence.

**Additional file 2. **Characteristics of included participants from the school employee, student, and parent group.

**Additional file 3. **Compliance to regular testing in relation to different measures, among students in primary-, lower and upper secondary school.

**Additional file 4. **Satisfaction level of contact tracing teams, school administrators and school employees in relation to their cooperation with different stakeholders involved in regular testing.

**Additional file 5. **Confidence in the implementation of regular testing by contact tracing teams, school administrators, school employees, students, and parents in Oslo and Viken.

**Additional file 6. **Employees in student-oriented work and their concern of transmitting infection after the implementation of regular testing, reported by school administrations in Oslo and Viken.

**Additional file 7. **Confidence in the decision to implement regular testing and absence from work by school employees in Oslo and Viken.

**Additional file 8. **Concerns regarding the implementation of regular testing and sociodemographic factors by parents and students in upper secondary school in Oslo and Viken.

**Additional file 9. **Concerns regarding the implementation of regular testing and sociodemographic factors by parents and students in upper secondary school in Oslo and Viken.

**Additional file 10. **Concerns regarding the implementation of regular testing and consequences by parents and students in upper secondary school in Oslo and Viken.

**Additional file 11. **Increased school attendance in relation to different indicators by parents and students in upper secondary school in Oslo and Viken.

**Additional file 12. **Compliance to regular testing in relation to gender or foreign origin among students in upper secondary school.

## Data Availability

The dataset used and/or analysed during the current study are available from the corresponding author upon reasonable request. This dataset is not made publicly available.

## Abbreviations

IPC: Infection prevention measures
NIPH: Norwegian Institute of Public Health
RT: Regular testing
