# Supplementary material for: Experiences with regular testing of students for SARS-CoV-2 in primary and secondary schools: results from a cross-sectional study in two Norwegian counties, autumn 2021
Source: BMC Public Health. 2023 Aug 15;23:1548. doi: 10.1186/s12889-023-16452-7 (PMC10426148; doi:10.1186/s12889-023-16452-7)
Supplement: Supplementary file 1 — Additional file 1. Number of participants included from the different groups by county of residence. [file 12889_2023_16452_MOESM1_ESM.docx]

Additional file 1. Number of participants included from the different groups by county of residence.

| **County** | **Contact tracing teams** | **School administrators** | **School employees** | **Students (upper- secondary)** | **Parents (primary and lower- secondary)** | **Total** |
| --- | --- | --- | --- | --- | --- | --- |
| Oslo | 24 | 33 | 133 | 59 | 1782 | 2031 |
| Viken | 6 | 51 | 247 | 991 | 1239 | 2534 |
| Total | 30 | 84 | 380 | 1050 | 3021 | 4565 |
