# Supplementary material for: Experiences with regular testing of students for SARS-CoV-2 in primary and secondary schools: results from a cross-sectional study in two Norwegian counties, autumn 2021
Source: BMC Public Health. 2023 Aug 15;23:1548. doi: 10.1186/s12889-023-16452-7 (PMC10426148; doi:10.1186/s12889-023-16452-7)
Supplement: Supplementary file 2 — Additional file 2. Characteristics of included participants from the school employee, student, and parent group. [file 12889_2023_16452_MOESM2_ESM.docx]

Additional file 2: Characteristics of included participants from the school employee, student, and parent group.

|  | **Sample group** | | |
| --- | --- | --- | --- |
| **Characteristics** | **School employees, N=380** | **Students (upper-secondary), N=1050** | **Parents (primary and lower-secondary), N=3021** |
| **Gender** |  |  |  |
| Female | 266 (70%) | 650 (62%) | 2235 (74%) |
| Male | 110 (29%) | 370 (35%) | 755 (25%) |
| Unknown | 4 (1%) | 30 (3%) | 31 (1%) |
| **Median age (years)** | 46 | 17 | 46 |
| **School type** |  |  |  |
| Primary school 1-4  Primary school 5-7 | 82* | -  - | 1002 (33%)  652 (22%) |
| Lower secondary school 8-10 | 129* | - | 1358 (45%) |
| Upper secondary school | 186* |  | - |
| First year | - | 507 (48%) | - |
| Second year | - | 426 (40%) | - |
| Third year | - | 115 (11%) | - |
| Other | - | <1% | <1% |
| **Degree program** |  |  |  |
| Preparation for college/universities | - | 844 (80%) | - |
| Vocational studies | - | 181 (17%) | - |
| Other | - | 25 (3%) | - |
| **Educational level** |  |  |  |
| None | - | - | 2 (<1%) |
| Primary and lower secondary school | - | - | 45 (1.5%) |
| Upper secondary or vocational college | - | - | 358 (12%) |
| College | - | - | 165 (5.5%) |
| University lowest (four years or less) | - | - | 1023 (34%) |
| University (four years or more) | - | - | 1397 (46%) |
| Unknown | - | - | 31 |
| **Role** |  |  |  |
| Teachers | 309 (81%) | - | - |
| Other student-oriented roles (child welfare workers, physical therapist, social worker | 46 (12%) | - | - |
| Other (librarians, janitors) | 25 (7%) | - | - |
| **Country of birth** |  |  |  |
| Norway | 348 (92%) | 935 (90%) | 2562 (85%) |
| Foreign country | 24 (6%) | 115 (10%) | 459 (15%) |
| Unknown | 8 (2%) | - | - |

^*^More than one response could be selected hence no percentage calculation
