# Supplementary material for: Experiences with regular testing of students for SARS-CoV-2 in primary and secondary schools: results from a cross-sectional study in two Norwegian counties, autumn 2021
Source: BMC Public Health. 2023 Aug 15;23:1548. doi: 10.1186/s12889-023-16452-7 (PMC10426148; doi:10.1186/s12889-023-16452-7)
Supplement: Supplementary file 3 — Additional file 3. Compliance to regular testing in relation to different measures, among students in primary-, lower and upper secondary school. [file 12889_2023_16452_MOESM3_ESM.docx]

Additional file 3: Compliance to regular testing in relation to different measures, among students in primary-, lower and upper secondary school

|  | **Parents (primary and lower-secondary), N=3021** | | | **Students (upper-secondary), N=1050** | | |
| --- | --- | --- | --- | --- | --- | --- |
| **Compliance in relation to:** | Compliant,  N=2801^1^ | Non-compliant,  N=220^1^ | p-value^2^ | Compliant,  N=970^1^ | Non-compliant,  N=80^1^ | p-value^2^ |
| **Age category** | | | **<0.001** |  |  | - |
| 15-30 | 15 (<1%) | 3 (1%) |  | - | - |  |
| 31-40 | 556 (20%) | 72 (33%) |  | - | - |  |
| 41-50 | 1780 (64%) | 120 (55%) |  | - | - |  |
| 51-60 | 423 (15%) | 24 (11%) |  | - | - |  |
| 61-80 | 27 (1%) | 1 (<1%) |  | - | - |  |
| **School grade** | | | **<0.001** |  |  | **0.045** |
| Primary school 1-4 | 897 (32%) | 105 (48%) |  | - | - |  |
| Primary school 5-7 | 597 (21%) | 55 (25%) |  | - | - |  |
| Other | 8 (<1%) | 1 (<1%) |  | 2 (<1%) | - |  |
| Lower secondary school 8-10 | 1299 (46%) | 59 (27%) |  | - | - |  |
| Upper secondary school |  |  |  |  |  |  |
| First year | - | - |  | 478 (49%) | 29 (36%) |  |
| Second year | - | - |  | 390 (40%) | 36 (45%) |  |
| Third year | - | - |  | 100 (10%) | 15 (19%) |  |
| **Weekly number of tests** |  |  | **<0.001** |  |  | **<0.001** |
| 1 | 249 (9%) | 137 (62%) |  | 77 (8%) | 47 (59%) |  |
| 2 | 2378 (85%) | 35 (16%) |  | 749 (77%) | 28 (35%) |  |
| 3 | 146 (5%) | 1 (<1%) |  | 144 (15%) | 0 |  |
| Unknown | 28 (1%) | 47 (21%) |  | - | 5 (6%) |  |
| **Testing place** |  |  | **<0.001** |  |  | **0.008** |
| At home | 1578 (56%) | 128 (58%) |  | 569 (59%) | 40 (50%) |  |
| At school | 391 (14%) | 14 (6%) |  | 49 (5%) | 2 (2.5%) |  |
| Test-station | 13 (<1%) | 9 (4%) |  | 0 | 1 (1%) |  |
| 1st at school, other tests at home | 602 (21%) | 31 (14%) |  | 293 (30%) | 28 (35%) |  |
| 1st at the test-station, other tests at home | 154 (5%) | 18 (8%) |  | 45 (5%) | 4 (5%) |  |
| Other | 44 (2%) | 18 (8%) |  | 5 (<1%) | 2 (2.5%) |  |
| Unknown | 19 (1%) | 2 (1%) |  | 9 (1%) | 3 (4%) |  |
| **Difficulties regarding regular testing** |  |  |  |  |  |  |
| Lack of training | 32 (1%) | 10 (4.5%) | **<0.001** | 37 (4%) | 7 (9%) | **0.072** |
| Lack of time | 158 (6%) | 39 (18%) | **<0.001** | 139 (14%) | 25 (31%) | **<0.001** |
| Lack of motivation | - | - | **-** | 95 (10%) | 24 (30%) | **<0.001** |
| Lack of information and communication | 61 (2%) | 10 (4.5%) | **0.026** | 57 (6%) | 9 (11%) | **0.057** |
| Lack of access to testing equipment | 133 (5%) | 15 (7%) | **0.2** | 86 (9%) | 9 (11%) | **0.5** |
| **Infection and Prevention Control measures** |  |  |  |  |  |  |
| Compliant with IPC-measures outside school | 2759 (99%) | 214 (97%) | **0.2** | 760 (78%) | 42 (52%) | **<0.001** |
| Compliance with IPC measures at school | - | - | - |  |  | **<0.001** |
| Yes |  |  |  | 806 (83%) | 43 (54%) |  |
| No |  |  |  | 139 (14%) | 34 (42%) |  |
| Unknown |  |  |  | 25 (3%) | 3 (4%) |  |
| **Confidence in the health authorities** |  |  | **<0.001** |  |  | - |
| Confident | 1943 (69%) | 110 (50%) |  | - | - |  |
| Not confident | 763 (27%) | 87 (39%) |  | - | - |  |
| Unknown | 95 (3%) | 23 (10.5%) |  | - | - |  |
| **Consider regular testing to be a safe alternative to quarantine** |  |  | **<0.001** |  |  |  |
| Yes | 2459 (88%) | 159 (72%) |  | - | - |  |
| No | 342 (12%) | 61 (28%) |  | - | - |  |
| **Negative experience with regular testing** |  |  | **<0.001** |  |  | **0.032** |
| Yes | 487 (17%) | 84 (38%) |  | 80 (8%) | 14 (17%) |  |
| No | 2258 (81%) | 124 (56%) |  | 839 (86%) | 63 (79%) |  |
| Unknown | 56 (2%) | 12 (6%) |  | 51 (5%) | 3 (4%) |  |

^1^ n (%)

^2^ Pearson´s Chi-squared test; Fisher´s exact test
