# Supplementary material for: Experiences with regular testing of students for SARS-CoV-2 in primary and secondary schools: results from a cross-sectional study in two Norwegian counties, autumn 2021
Source: BMC Public Health. 2023 Aug 15;23:1548. doi: 10.1186/s12889-023-16452-7 (PMC10426148; doi:10.1186/s12889-023-16452-7)
Supplement: Supplementary file 4 — Additional file 4. Satisfaction level of contact tracing teams, school administrators and school employees in relation to their cooperation with different stakeholders involved in regular testing. [file 12889_2023_16452_MOESM4_ESM.docx]

Additional file 4: Satisfaction level of contact tracing teams, school administrators and school employees in relation to their cooperation with different stakeholders involved in regular testing.

| **Contact tracing teams and cooperation with the school administration / educational authorities** | | |
| --- | --- | --- |
| *County* | *Oslo, N=24* | *Viken, N=6* |
| **Information flow** |  |  |
| Satisfied | 13 (54%) | 6 (100%) |
| Not satisfied | 8 (33%) |  |
| Unknown | 3 (13%) |  |
| **Role clarification** |  |  |
| Satisfied | 15 (63%) | 4 (67%) |
| Not satisfied | 6 (25%) | 2 (33%) |
| Unknown | 3 (13%) |  |
| **Availability of testing equipment** |  |  |
| Satisfied | 14 (58%) | 6 (100%) |
| Not satisfied | 2 (8%) |  |
| Unknown | 8 (33%) |  |
| **Guidance on how to conduct regular testing** |  |  |
| Satisfied | 13 (54%) | 5 (83%) |
| Not satisfied | 1 (4%) | 1 (17%) |
| Unknown | 10 (42%) |  |
| **School administrators and cooperation with the municipality** | | |
| *County* | *Oslo, N = 33* | *Viken, N = 51* |
| **Information flow** |  |  |
| Satisfied | 30 (91%) | 41 (80%) |
| Not satisfied | 2 (6%) | 7 (14%) |
| Unknown | 1 (3%) | 3 (6%) |
| **Role clarification** |  |  |
| Satisfied | 24 (73%) | 32 (63%) |
| Not satisfied | 8 (24%) | 11 (22%) |
| Unknown | 1 (3%) | 8 (16%) |
| **Availability of testing equipment** |  |  |
| Satisfied | 30 (91%) | 41 (80%) |
| Not satisfied | 2 (6%) | 9 (18%) |
| Unknown | 1 (3%) | 1 (2%) |
| **Guidance on how to conduct regular testing** |  |  |
| Satisfied | 30 (91%) | 41 (80%) |
| Not satisfied | 2 (6%) | 6 (12%) |
| Unknown | 1 (3%) | 4 (8%) |
| **School employees and cooperation with the school administrators** | | |
| *County* | *Oslo, N = 133* | *Viken, N = 247* |
| **Information flow** |  |  |
| Satisfied | 96 (72%) | 202 (82%) |
| Not satisfied | 23 (17%) | 17 (7%) |
| Unknown | 14 (11%) | 28 (11%) |
| **Role clarification** |  |  |
| Satisfied | 81 (61%) | 171 (69%) |
| Not satisfied | 31 (23%) | 32 (13%) |
| Unknown | 21 (16%) | 44 (18%) |
| **Availability of testing equipment** |  |  |
| Satisfied | 115 (86%) | 215 (87%) |
| Not satisfied | 13 (10%) | 7 (3%) |
| Unknown | 5 (4%) | 25 10%) |
| **Guidance on how to conduct regular testing** |  |  |
| Satisfied | 82 (62%) | 188 (76%) |
| Not satisfied | 33 (25%) | 32 (13%) |
| Unknown | 18 (14%) | 27 (11%) |
