# Supplementary material for: Experiences with regular testing of students for SARS-CoV-2 in primary and secondary schools: results from a cross-sectional study in two Norwegian counties, autumn 2021
Source: BMC Public Health. 2023 Aug 15;23:1548. doi: 10.1186/s12889-023-16452-7 (PMC10426148; doi:10.1186/s12889-023-16452-7)
Supplement: Supplementary file 5 — Additional file 5. Confidence in the implementation of regular testing by contact tracing teams, school administrators, school employees, students, and parents in Oslo and Viken. [file 12889_2023_16452_MOESM5_ESM.docx]

Additional file 5. Confidence in the implementation of regular testing by contact tracing teams, school administrators, school employees, students, and parents in Oslo and Viken.

|  | **Contact tracing teams, N=30** | | **School administrators, N=84** | | **School employees,**  **N=380** | | **Parents (primary and lower-secondary),** **N=3021** | | |
| --- | --- | --- | --- | --- | --- | --- | --- | --- | --- |
|  | Confident N=22^1^ | Non confident N=8^1^ | Confident N=81^1^ | Non confident N=3^1^ | Confident N=302^1^ | Non confident N=78^1^ | Confident N= 2053^1^ | Non confident, N=968^1^ | p-value^2^ |
| **County** | | | | | | | | | **0.5** |
| Oslo | 19 (79%) | 5 (21%) | 31 (94%) | 2 (6%) | 103 (77%) | 30 (22%) | 1220 (59%) | 562 (58%) |  |
| Viken | 3 (50%) | 3 (50%) | 50 (98%) | 1 (2%) | 199 (81 %) | 48 (19%) | 833 (41%) | 406 (42%) |  |
| **Age category** | | | | |  | | | | **0.034** |
| ≤30 | - | - | - | - | 37 (12%) | 5 (6%) | 13 (<1%) | 5 (<1%) |  |
| 31-40 | - | - | - | - | 73 (24%) | 30 (38%) | 397 (19%) | 231 (24%) |  |
| 41-50 | - | - | - | - | 75 (25%) | 23 (29%) | 1309 (64%) | 591 (61%) |  |
| 51-60 | - | - | - | - | 86 (28%) | 17 (22%) | 311 (15%) | 136 (14%) |  |
| ≥61 | - | - | - | - | 31 (10%) | 3 (4%) | 23 (1%) | 5 (<1%) |  |
| **Workplace** | | | | | | | | | - |
| Primary school | - | - | - | - | 62 (76%) | 20 (24%) | - | - |  |
| Lower secondary school | - | - | - | - | 98 (76%) | 31 (24%) | - | - |  |
| Upper secondary school | - | - | - | - | 155 (83%) | 31 (17%) | - | - |  |
| **Education level** | | | | | | | | | **0.007** |
| None | - | - | - | - | - | - | 2 (<0.1%) | 0 |  |
| Primary and secondary | - | - | - | - | - | - | 28 (1%) | 17 (2%) |  |
| Upper secondary | - | - | - | - | - | - | 230 (11%) | 128 (13%) |  |
| College | - | - | - | - | - | - | 96 (5%) | 69 (7%) |  |
| University ≤4 years | - | - | - | - | - | - | 691 (34%) | 332 (34%) |  |
| University ≥4 years | - | - | - | - | - | - | 988 (48%) | 409 (42%) |  |
| Unknown |  |  |  |  |  |  | 18 (<1%) | 13 (1%) |  |
|  | | | | | | | | | |

^1^n (%)

### ^2^Fisher's exact test
