# Supplementary material for: Experiences with regular testing of students for SARS-CoV-2 in primary and secondary schools: results from a cross-sectional study in two Norwegian counties, autumn 2021
Source: BMC Public Health. 2023 Aug 15;23:1548. doi: 10.1186/s12889-023-16452-7 (PMC10426148; doi:10.1186/s12889-023-16452-7)
Supplement: Supplementary file 6 — Additional file 6. Employees in student-oriented work and their concern of transmitting infection after the implementation of regular testing, reported by school administrations in Oslo and Viken. [file 12889_2023_16452_MOESM6_ESM.docx]

Additional file 6. Employees in student-oriented work and their concern of transmitting infection after the implementation of regular testing, reported by school administrations in Oslo and Viken.

| **Have employees in student-oriented work been concerned about transmitting infection after implementation of** regular testing**?** | Yes, N=32 | No, N=49 | Unknown |
| --- | --- | --- | --- |
| Oslo | 12 (36%) | 20 (61%) | <1% |
| Viken | 20 (39%) | 29 (57%) | <1% |
| Total | 32 (38%) | 49 (58%) | 5 (4%) |
