# Supplementary material for: Experiences with regular testing of students for SARS-CoV-2 in primary and secondary schools: results from a cross-sectional study in two Norwegian counties, autumn 2021
Source: BMC Public Health. 2023 Aug 15;23:1548. doi: 10.1186/s12889-023-16452-7 (PMC10426148; doi:10.1186/s12889-023-16452-7)
Supplement: Supplementary file 7 — Additional file 7. Confidence in the decision to implement regular testing and absence from work by school employees in Oslo and Viken. [file 12889_2023_16452_MOESM7_ESM.docx]

Additional file 7. Confidence in the decision to implement regular testing and absence from work by school employees in Oslo and Viken.

| **Sometimes stayed at home due to concern of transmitting COVID-19** | | | |
| --- | --- | --- | --- |
|  | Confident, N=302^1^ | Non confident, N=78^1^ | p-value^2^ |
|  |  |  | **0.017** |
| Agree | 11 (4%) | 7 (9%) |  |
| Disagree | 257 (85%) | 56 (72%) |  |
| Missing | 34 (11%) | 15 (19%) |  |

^1^n (%)

^1^Pearsons Chi-squared test
