# Supplementary material for: Experiences with regular testing of students for SARS-CoV-2 in primary and secondary schools: results from a cross-sectional study in two Norwegian counties, autumn 2021
Source: BMC Public Health. 2023 Aug 15;23:1548. doi: 10.1186/s12889-023-16452-7 (PMC10426148; doi:10.1186/s12889-023-16452-7)
Supplement: Supplementary file 8 — Additional file 8. Concerns regarding the implementation of regular testing and sociodemographic factors by parents and students in upper secondary school in Oslo and Viken. [file 12889_2023_16452_MOESM8_ESM.docx]

Additional file 8: Concerns regarding the implementation of regular testing and sociodemographic factors by parents and students in upper secondary school in Oslo and Viken.

| **Parents (primary and lower-secondary), N=2411 (/3 021)** | | | | **Students (upper-secondary), 821 (/1 050)** | | |
| --- | --- | --- | --- | --- | --- | --- |
| **Have you been concerned that your child would become infected at school after regular testing was implemented?** | | | p-value^2^ | **Were you less concerned of becoming infected at school after regular testing was implemented?** | | p-value^2^ |
|  | Yes, N=950^1^ | No, N=1461^1^ |  | Yes, N=619^1^ | No, N=202^1^ |  |
| **Age category** |  |  | **0.5** |  |  | - |
| 15-30 | 6 (<1%) | 9 (<1%) |  | - | - |  |
| 31-40 | 196 (21%) | 296 (20%) |  | - | - |  |
| 41-50 | 593 (62%) | 946 (65%) |  | - | - |  |
| 51-60 | 149 (16%) | 196 (13%) |  | - | - |  |
| 61-80 | 6 (<1%) | 14 (1%) |  | - | - |  |
| **School grade** |  |  | **0.3** |  |  | **0.014** |
| Primary-school 1-4 | 293 (31%) | 493 (34%) |  | - | - |  |
| Primary-school 5-7 | 208 (22%) | 329 (23%) |  | - | - |  |
| Lower-secondary school 8-10 | 447 (47%) | 634 (43%) |  | - | - |  |
| Other | 2 (<1%) | 5 (<1%) |  | 0 (0%) | 2 (1%) |  |
| Upper-secondary school |  |  |  |  |  |  |
| First year | - | - |  | 297 (48%) | 90 (45%) |  |
| Second year | - | - |  | 244 (39%) | 94 (47%) |  |
| Third year | - | - |  | 78 (13%) | 16 (8%) |  |
| **County** |  |  | **0.005** |  |  | **0.4** |
| Oslo | 528 (56%) | 896 (61%) |  | 35 (6%) | 15 (7%) |  |
| Viken | 422 (44%) | 565 (39%) |  | 584 (94%) | 187 (93%) |  |
| **Educational level** |  |  | **<0.001** |  |  | **-** |
| None |  | 1 (<0.1%) |  | - | - |  |
| Primary and secondary | 21 (2%) | 14 (1%) |  | - | - |  |
| Upper secondary | 131 (14%) | 147 (10%) |  | - | - |  |
| Vocational school | 60 (6%) | 73 (5%) |  | - | - |  |
| University ≤4 years | 320 (34%) | 486 (33%) |  | - | - |  |
| University ≥4 years | 406 (43%) | 729 (50%) |  | - | - |  |
| Unknown | 12 (1%) | 11 (<1%) |  | - | - |  |
| **Study pathway** |  |  | - |  |  | **0.8** |
| Preparation for high schools and universities | - | - |  | 506 (82%) | 163 (81%) |  |
| Vocational studies | - | - |  | 97 (16%) | 35 (17%) |  |
| Other | - | - |  | 16 (2%) | 4 (2%) |  |

^1^n (%)

^2^ Fisher's exact test; Pearson's Chi-squared test
