# Supplementary material for: Experiences with regular testing of students for SARS-CoV-2 in primary and secondary schools: results from a cross-sectional study in two Norwegian counties, autumn 2021
Source: BMC Public Health. 2023 Aug 15;23:1548. doi: 10.1186/s12889-023-16452-7 (PMC10426148; doi:10.1186/s12889-023-16452-7)
Supplement: Supplementary file 10 — Additional file 10. Concerns regarding the implementation of regular testing and consequences by parents and students in upper secondary school in Oslo and Viken. [file 12889_2023_16452_MOESM10_ESM.docx]

Additional file 10: Concerns regarding the implementation of regular testing and consequences by parents and students in upper secondary school in Oslo and Viken.

| **Parents (primary and lower-secondary), N = 2411 (/3 021)** | | | | **Students (upper-secondary), N = 821 (/1 050)** | | | |
| --- | --- | --- | --- | --- | --- | --- | --- |
|  | **Have you been concerned that your child would become infected at school after regular testing was implemented?** | | p-value^2^ |  | **Were you less concerned of becoming infected at school after regular testing was implemented?** | | p-value^2^ |
|  | Yes, N=950^1^ | No, N=1461^1^ |  |  | Yes, N=619^1^ | No, N=202^1^ |  |
| **Sometimes kept my child at home to protect my child from becoming infected** |  |  | **<0.001** |  |  |  | - |
| Agree | 180 (19%) | 30 (2%) |  |  | - | - |  |
| Disagree | 689 (72%) | 1418 (97%) |  |  | - | - |  |
| Unknown | 81 (9%) | 13 (1%) |  |  | - | - |  |
| **Sometimes kept my child at home to protect myself or other household members from becoming infected** |  |  | **<0.001** | **Sometimes stayed at home to protect myself or other household members from becoming infected** |  |  | **0.2** |
| Agree | 152 (16%) | 25 (2%) |  | Agree | 183 (30%) | 55 (27%) |  |
| Disagree | 721 (76%) | 1415 (96%) |  | Disagree | 323 (52%) | 125 (62%) |  |
| Unknown | 77 (8%) | 21 (2%) |  | Unknown | 113 (18%) | 22 (11%) |  |
| **Has your child participated in extracurricular activities?** |  |  | **<0.001** | **Have you participated in extracurricular activities?** |  |  | **0.7** |
| Yes | 468 (49%) | 939 (64%) |  | Yes | 543 (88%) | 175 (87%) |  |
| No | 482 (51 %) | 522 (36 %) |  | No | 76 (12%) | 27 (13%) |  |
| **Test compliance** |  |  | **0.5** | **Test compliance** |  |  | **0.002** |
| Compliant | 878 (92%) | 1 360 (93%) |  | Compliant | 585 (95%) | 178 (88%) |  |
| Non- compliant | 72 (8%) | 101 (7%) |  | Non- compliant | 34 (5%) | 24 (12%) |  |

^1^n (%)

^2^Pearson's Chi-squared test
