# Supplementary material for: Experiences with regular testing of students for SARS-CoV-2 in primary and secondary schools: results from a cross-sectional study in two Norwegian counties, autumn 2021
Source: BMC Public Health. 2023 Aug 15;23:1548. doi: 10.1186/s12889-023-16452-7 (PMC10426148; doi:10.1186/s12889-023-16452-7)
Supplement: Supplementary file 11 — Additional file 11. Increased school attendance in relation to different indicators by parents and students in upper secondary school in Oslo and Viken. [file 12889_2023_16452_MOESM11_ESM.docx]

Additional file 11: Increased school attendance in relation to different indicators by parents and students in upper secondary school in Oslo and Viken

|  | **Parents (primary and lower-secondary), N= 2042 (/3021)** | |  | **Students (upper-secondary), N = 617 (/1 050**) | | |
| --- | --- | --- | --- | --- | --- | --- |
| Increased school attendance | Yes, N=1591^1^ | No, N=451^1^ | p-value^2^ | Yes, N=450^1^ | No, N=167^1^ | p-value^2^ |
| **Test compliance** |  |  | **<0.001** |  |  | **0.017** |
| Compliant  Not compliant | 1498 (94%)  93 (5.8%) | 397 (88%)  54 (12%) |  | 426 (95%)  24 (5.3%) | 147(88%)  20(12%) |  |
| **Confidence** |  |  | **<0.001** |  |  | - |
| Confident  Not confident | 1172 (74%)  419 (26%) | 229 (51%)  222 (49%) |  | -  - | -  - |  |
| **Satisfied with the organisation** |  |  | **<0.001** |  |  | **<0.001** |
| Yes  No  Unknown | 1417 (89%)  41 (3%)  133 (8%) | 287 (64%)  77 (17%)  87 (19%) |  | 336 (75%)  14 (3%)  100 (22%) | 76 (45%)  31 (19%)  60 (36%) |  |
| **Satisfied with the information provided (general)** |  |  | - |  |  | **<0.001** |
| Yes  No | -  - | -  - |  | 330 (96%)  20 (5.7%) | 91 (78%)  25 (22%) |  |
| **Satisfied with the information provided by the school** |  |  | **<0.001** |  |  | - |
| Yes  No | 1401 (88%)  45 (3%) | 320 (71%)  47 (10%) |  | -  - | -  - |  |
| Unknown | 145 (9%) | 84 (19%) |  |  |  |  |
| **Satisfied with the information provided by the municipality** |  |  | **<0.001** |  |  | - |
| Yes  No | 1099 (69%)  90 (6%) | 190 (42%)  87 (19%) |  | -  - | -  - |  |
| Unknown | 402 (25%) | 174 (39%) |  |  |  |  |
| **Lack of time** | 78 (4.9%) | 49 (11%) | **<0.001** | 67 (15%) | 35 (21%) | **0.11** |
| **Lack of training** | 17 (1.1%) | 10 (2.2%) | **0.2** | - | - | - |
| **Lack of motivation** | - | - | - | 43 (9.6%) | 26 (16%) | **0.11** |

^1^n (%)

^2^Pearson's Chi-squared test
