# Supplementary material for: Experiences with regular testing of students for SARS-CoV-2 in primary and secondary schools: results from a cross-sectional study in two Norwegian counties, autumn 2021
Source: BMC Public Health. 2023 Aug 15;23:1548. doi: 10.1186/s12889-023-16452-7 (PMC10426148; doi:10.1186/s12889-023-16452-7)
Supplement: Supplementary file 12 — Additional file 12. Compliance to regular testing in relation to gender or foreign origin among students in upper secondary school. [file 12889_2023_16452_MOESM12_ESM.docx]

Additional file 12: Compliance to regular testing in relation to gender or foreign origin among students in upper secondary school

|  | **Students (upper-secondary), N=1050** | | |
| --- | --- | --- | --- |
| **Compliance in relation to:** | Compliant,  N=970^1^ | Non-compliant,  N=80^1^ | p-value^2^ |
| **Gender** |  |  | **0.09** |
| Female | 609 (63%) | 41 (51%) |  |
| Male | 336 (35%) | 34 (42%) |  |
| Unknown | 25 (2%) | 5 (6%) |  |
| **Both parents born in Norway** |  |  | **0.6** |
| Yes | 713 (74%) | 58 (72%) |  |
| No | 242 (25%) | 20 (25%) |  |
| Do not want to answer | 10 (1%) | 2 (2%) |  |
| Do not know | 5 (<1%) | - |  |

### ^1^n (%)

### ^2^ Pearson's Chi-squared test; Fisher's exact test
